# Fetal Health Diagnosis Based on Adaptive Dynamic Weighting with Main-Auxiliary Correction Network

**DOI:** 10.3390/biotech14030057

**Published:** 2025-07-28

**Authors:** Haiyan Wang, Yanxing Yin, Liu Wang, Yifan Wang, Xiaotong Liu, Lijuan Shi

**Affiliations:** 1 College of Computer Science and Technology, Changchun University, Changchun 130022, China; yinyanxingstruggle@163.com (Y.Y.); wangl95@ccu.edu.cn (L.W.); wangyf94@ccu.edu.cn (Y.W.); liuxiaotonglucky@163.com (X.L.); 2 College of Electronic Information Engineering, Changchun University, Changchun 130012, China

**Keywords:** fetal health, cardiotocography (CTG), data category imbalance, adaptive weighting, secondary correction

## Abstract

Maternal and child health during pregnancy is an important issue in global public health, and the classification accuracy of fetal cardiotocography (CTG), as a key tool for monitoring fetal health during pregnancy, is directly related to the effectiveness of early diagnosis and intervention. Due to the serious category imbalance problem of CTG data, traditional models find it challenging to take into account a small number of categories of samples, increasing the risk of leakage and misdiagnosis. To solve this problem, this paper proposes a two-step innovation: firstly, we design a method of adaptive adjustment of misclassification loss function weights (MAAL), which dynamically identifies and increases the focus on misclassified samples based on misclassification rates. Secondly, a primary and secondary correction network model (MAC-NET) is constructed to carry out secondary correction for the misclassified samples of the primary model. Experimental results show that the method proposed in this paper achieves 99.39% accuracy on the UCI publicly available fetal health dataset, and also obtains excellent performance on other domain imbalance datasets. This demonstrates that the model is not only effective in alleviating the problem of category imbalance, but also has very high clinical utility.

## 1. Introduction

Maternal and infant health during pregnancy is an important area of research in global public health [1]. According to the World Health Organization (WHO), approximately more than 4.5 million women and infants have died each year during pregnancy, childbirth, or the first weeks of life since 2015, which is equivalent to one death every seven seconds. Most of these are due to preventable complications such as intrauterine distress, hypoxia, or preterm delivery [2]. Misdiagnosis and underdiagnosis significantly exacerbate the risk: studies have shown a 68% rate of misdiagnosis in referral centers, which directly contributes to a maternal mortality rate of 6.2% (13 deaths) and a perinatal mortality rate of 461 per 1000 live births [3]. Cardiotocography (CTG) is one of the most commonly used means of monitoring fetal health in clinical practice. By recording fetal heart rate (FHR) and uterine contraction (UC) signals, physicians can assess the intrauterine condition of the fetus [4]. However, the interpretation of CTG patterns is highly dependent on physicians’ experience, and there are often significant differences in the judgment of different physicians on the same pattern, which may also lead to misdiagnosis or omission of diagnosis [5]. Doctors usually classify CTG according to the criteria of the International Federation of Gynecology and Obstetrics (FIGO) [6], including normal, suspicious, and pathological classifications. However, in practice, there is a significant category imbalance in CTG data, in which the samples in the pathological category are relatively sparse, leading to insufficient identification of a few categories by the model, thus increasing the risk of missed diagnosis [7,8]. In addition, CTG signals are greatly affected by the external environment and individual differences, and feature overlap and boundary ambiguity often occur, making the model prone to miscalculation when dealing with these “difficult samples.” Additionally, misdiagnosis occurs in the clinic, affecting the timeliness of clinical interventions, while, at the same time, there are limitations in the existing model in terms of feature extraction and dynamic weighting. At the same time, the existing models have limitations in feature extraction and dynamic weighting, which make it difficult to fully explore the potential information in CTG data, limiting the improvement of diagnostic performance. Therefore, intelligent diagnosis based on CTG data has become an important direction of current fetal health research [9]. Given the above problems, it is particularly urgent to construct a deep learning-based auxiliary diagnosis system for fetal health. Such systems can not only realize the rapid identification of fetal health status and provide objective decision support for clinicians, thus improving the accuracy of CTG monitoring and the effectiveness of clinical interventions [10], but also help to identify high-risk fetuses as early as possible in the prenatal stage, prompting doctors to take interventions in advance to reduce the fetal mortality rate and improve the health of mothers and infants [2].

To address the problem of fetal health diagnosis, in recent years, scholars have proposed a variety of methods based on machine learning (ML) and deep learning (DL) to improve the accuracy and stability of assisted diagnosis of fetal health and have made some progress in the task of CTG data classification. In ML, Shastry K.A. et al. [11] proposed AWD model combining feature selection with an adaptive weighting mechanism, Sahin H. et al. [12] evaluated the classification performance of eight ML models, Pradhan A K et al. [13] optimized hyperparameters to achieve 99% accuracy on CTG data through random forest (RF). Akbulut A [14] used the integration of an e-health application with ML technology to build a prenatal anomaly prediction system based on the data of 96 pregnant women, while Kuzu A. et al. [15] designed an integrated learning model to achieve 99.5% accuracy on NST data and Chen Y et al. [16] proposed a deep forest (DF) algorithm to achieve 92.64% accuracy on the CTG dataset. Bharadwaj K [17] studied and proposed an active learning technique based on uncertainty and diversity criteria to achieve over 99% accuracy in the UCI fetal health dataset with only 20% training data. In terms of DL, Mushtaq G. et al. [18] achieved 99% accuracy on the CTG dataset of UCI based on a deep neural network (DNN); Ye Fei Z. [19] used BLSTM combined with MFCC feature extraction for fetal distress diagnosis; Deng Y et al. [20] designed a lightweight LW-FHR net on the CTU-UHB database to achieve 95.24% accuracy; and Muhammad Hussain N. et al. [21] combined AlexNet and SVM to construct a lightweight model and achieved 99.72% accuracy on the CTG dataset of UCI. Cömert Z. et al. [22] investigated a comparison of feedforward neural networks (ANN) with extreme learning machines (ELMs) on the UCI database ELM outperformed ANN with 93.42% accuracy on fetal health data. Although the above studies have made some progress in the task of classifying CTG data, these methods still have shortcomings in dealing with extremely unbalanced data, which leads to limited generalization of the models to complex cases. Therefore, further optimization of the algorithms is still needed to enhance the recognition ability of a few types of samples, reduce the risk of misclassification, and improve the clinical applicability of CTG-assisted diagnosis systems.

For the category imbalance problem, scholars have proposed a variety of optimization strategies, which can be mainly classified into two categories of data-level and algorithm-level methods. Among these, the data level methods include oversampling and undersampling. In terms of oversampling, Ilham A et al. [7] used CFCM combined with SMOTE to deal with the imbalance problem, Dutta P et al. [23] utilized SMOTE combined with five ML models such as random forest (RF) to solve the imbalance problem and in turn to improve the prediction accuracy, and Al Duhayyim M [24] used randomized oversampling (ROS) and combined XGBoost, LGBM and CatBoost to construct an integrated model to achieve 99% classification accuracy in the CTG dataset; in addition, the Smote-XGBoost framework proposed by Yang J [25] and the Stacking model constructed by Wang X [26] et al., combining SMOTE and cost-sensitive learning, have also achieved good results in optimizing data distribution. The undersampling aspect is also widely used, Goyal S [27] proposed the neighborhood-based undersampling algorithm (N-US) to achieve the highest AUC (95.6%) and accuracy (96.9%) on the NASA software defect prediction dataset, while Bai L’s [28] two-step integrated undersampling algorithm (TSSE-BIM) effectively mitigates class overlap through a two-stage balanced undersampling and weighted boundary sampling process, which effectively mitigates the information loss in class overlapping scenarios. At the algorithmic level, there are mainly two types of methods based on focal loss and dynamic weighted loss for the category imbalance problem. For focal loss, Shi W [29] proposed a PFPS intelligent diagnosis model combining a 1D CNN with focal loss. Zhang Y [30] utilized a bidirectional multiplexed CNN with time-frequency feature fusion and introduced the focal loss and multi-task balancing and recalibration neural network (MTBR-Net) proposed by Sun W [31], both of which significantly improve the model’s focus on minority class samples; while, for dynamic weighted loss, Fernando K R M [32] proposed the dynamic weighted balanced loss function (DWB) to achieve intensive training of difficult samples by fusing class frequency and prediction probability, Li Z [33] introduced dynamic weighted entropy (DWE) criterion for e-fraud detection, Dong Y [34] used dynamic exponentially weighted cross-entropy loss in a hydroacoustic dataset to significantly improve the recognition rate, and Hu K [35] constructed a joint optimization framework based on a multi-scale deep supervised network with adaptive auxiliary loss (DSN-SAAL). Each of these methods has its advantages, but all of them have their limitations, which provide research space to further improve the model performance under unbalanced data.

**Figure 1:**
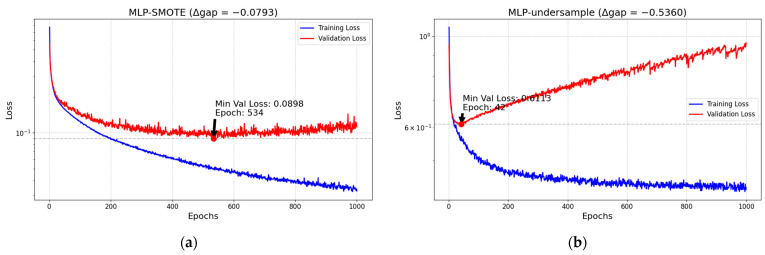
Training and validation loss comparison plots: (**a**) MLP-SMOTE comparison plot (Δ gap = −0.0793) and (**b**) MLP-undersample comparison plot (Δ gap = −0.5360).

Although data-level oversampling and undersampling methods alleviate the category imbalance problem to some extent, oversampling may introduce redundant data and lead to model overfitting [36,37], while undersampling may cause information loss and affect the overall model performance. In this paper, based on the MLP model shown in Figure 1, the training loss and validation loss of both SMOTE (oversampling) and undersampling strategies are validated for overfitting, and the Δ gap of the two at the end epoch is quantified. Although the Δ gap of the SMOTE method (Figure 1a) is relatively small, it can be seen clearly that the training loss continues to decrease while the validation loss shows an upward trend, which suggests that there is a tendency towards overfitting in the extended data of the model. Extended data tend to overfit. In the under-sampling method (Figure 1b), due to the removal of a large number of samples, the model is more prone to overfitting, and not only does Δ gap increase significantly, but the validation loss also rises along with the training process, which further verifies that the under-sampling strategy poses a serious overfitting problem. In addition, although methods such as focal loss at the algorithmic level can enhance the focus on a small number of class samples, their static weight adjustment approach lacks adaptivity to the data distribution and still relies on manual parameter adjustment, making it difficult to achieve dynamic optimization in complex medical data.

**Figure 2:**
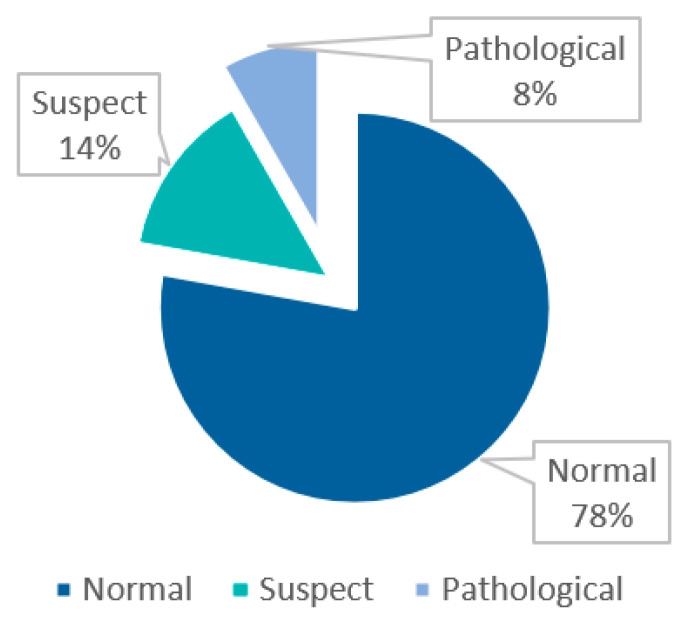
Distribution of CTG data.

To address these problems, this paper analyzes the serious imbalance of the category distribution based on the fetal health CTG dataset provided by UCI, as shown in Figure 2. To improve the ability to recognize samples of a few categories and at the same time reduce the risk of misdiagnosis and omission, this paper designs an adaptive adjustment loss function weight algorithm, misclassification-aware adaptive loss (MAAL), based on misclassification. The algorithm can dynamically adjust the loss weight according to the misclassification situation so that the model pays more attention to the difficult-to-classify samples during the training process. The accuracy of a single model may still be affected by the category imbalance, which leads to some complex samples being difficult to classify correctly. This paper further designs a main-auxiliary correction network (MAC-Net). Based on the primary model, a secondary correction model is introduced to relearn the misclassified samples and improve the model’s ability to recognize a few classes. MAC-Net optimizes the classification results through a dynamic weight adjustment mechanism, which enables the model to show stronger generalization ability in complex and unbalanced medical datasets and significantly reduces the risk of misdiagnosis and underdiagnosis. The experimental results show that the method has high clinical application value in improving the classification performance of CTG data.

The contributions of this paper will be briefly described in the following four aspects:

(1) Proposing a modeling framework for the dynamic main-auxiliary correction network (MAC-NET).
(2) Designing a misclassification-aware adaptive loss algorithm (MAAL) for the case of category imbalance.
(3) Developing a multi-granularity classification module based on multi-head attention fusion adaptive adjustment of misclassification weights.
(4) Designing an error sample management mechanism to accurately extract the primary model classification error samples and prevent the data leakage from causing the model effect to be inflated.
(5) The method proposed in this paper effectively mitigates the problem of poor accuracy and misdiagnosis and omission caused by the common data category imbalance phenomenon in the field of AI-assisted diagnosis and has been validated to achieve an accuracy of 99.39% on publicly available fetal health datasets.

## 2. Materials and Methods

### 2.1. Model Design and Realization

**Figure 3:**
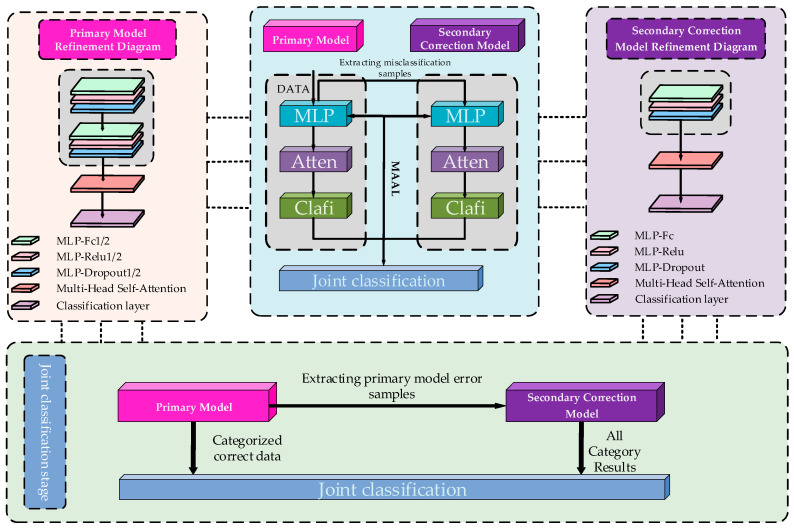
MAC-NET modeling framework diagram.

In the primary model phase, we use (shown in the upper left of Figure 3) the multi-layer perceptron (MLP, implemented in PyTorch 1.8.0, Facebook AI Research, Menlo Park, CA, USA)) to mine the nonlinear relationships between features and combine it with the MAAL to alleviate the problem of category imbalance by adjusting the weights of the loss functions of different categories through backpropagation. To further enhance the model’s ability to express features, the multi-head attention (MHA, implemented in PyTorch 1.8.0, Facebook AI Research, Menlo Park, CA, USA) mechanism is introduced, which enables the model to focus more precisely on the key features of a few classes of samples by assigning different attention weights to the features, thus optimizing the classification performance in the case of class imbalance. In addition, the MAC-NET framework incorporates a combination of coarse-grained and fine-grained classification strategies to enhance the model’s adaptability and classification ability. At the coarse-grained level, the adaptive weighting strategy can effectively balance the learning ability between different categories; at the fine-grained level, the multi-head self-attention mechanism plays an important role in intra-class feature aggregation and key feature extraction. The method can not only effectively alleviate the class imbalance problem but also improve the overall classification performance of the model.

In the secondary correction model stage, although the primary model achieves better classification results in the category imbalance scenario, there are still some misclassification phenomena in the identification of complex samples and decision boundary samples. For this reason, this paper proposes a secondary correction model (shown in the upper right of Figure 3) to recorrect the misclassified samples of the primary model to further improve the classification ability of the model. In the stage of the secondary correction model, the misclassification samples of the primary model are first extracted during its training process and used as the training data of the secondary correction model. At the same time, the misclassification samples of the primary model on the testing stage are collected as the test data of the secondary correction model. To prevent data leakage, a data leakage detection mechanism is designed in this paper to ensure that there are no overlapping samples between the extracted training and testing data to avoid the problem of inflated performance or overfitting of the secondary correction model. In addition, as the class imbalance problem still exists in the misclassified samples, the secondary correction model adopts the same MAAL combined with the multi-head self-attention mechanism method as the primary model. To ensure the learning ability of the secondary correction model on a few class samples, it can recognize complex samples more effectively and further improve the classification performance of the model on complex samples.

In the joint classification phase, the MAC-NET framework imports the optimal model obtained from the training of the primary model and the secondary correction model into the dynamic joint classification module. First, the input test samples are initially classified by the primary model. For the samples misclassified by the primary model, they are passed as input to the secondary correction model for reclassification. Finally, the classification results of the secondary correction model and the correctly predicted results of the primary model are combined, and the final results are obtained.

### 2.2. Multilayer Perceptron

A multilayer perceptron (MLP) is an artificial neural network consisting of multiple layers of neurons, which are arranged in a hierarchical structure. MLPs work on the principle of backpropagation, a key step in the training process. In the backpropagation process, the network adjusts its weights and biases by backpropagating errors from the output layer to the input layer. The iterative process allows for the fine-tuning of the parameters of the model so that it can make more accurate predictions over time.

The MLP algorithm consists of the following main components. The input layer receives input data and passes it to the hidden layer. Its number of neurons is equal to the dimension of the input features. The hidden layer consists of one or more neurons to transform the input data. The network performance is optimized by adjusting the number of hidden layers and neurons in each layer. The activation function performs a nonlinear transformation on the output of each neuron in the hidden layer. Common activation functions include Sigmoid, ReLU, Tanh, etc. The output layer is the final output of the network, and includes classification labels or regression targets. The number of neurons in the output layer depends on the specific data, e.g., the number of categories in a classification problem.

In this paper, the MLP in the primary model of the MAC-NET framework adopts the structure of input, ReLU, dropout, and hidden and output layers, while the difference between the secondary correction model and the primary model lies in the design of the number of layers of the MLP, adopting the input, ReLU, dropout, and output layers that are more lightweight compared with the primary model (see Figure 3 for the specific distribution). In this paper, the MAC-NET framework is configured with reasonable parameters (see Table 1) to improve its training efficiency.

### 2.3. Misclassification-Aware Adaptive Loss

#### 2.3.1. Cross Entropy Loss Function

Cross-entropy loss (CEL) is a widely used loss function for classification tasks, and its core idea is to measure the difference between the true category probability distribution and the model-predicted probability distribution and use it to guide the direction of model optimization. The goal is to minimize the cross-entropy so that the predicted probability distribution of the model is as close as possible to the true category distribution. The method originates from the information entropy theory, and the core idea is to optimize the model decision by maximizing the information gain. The mathematical expression of the cross-entropy loss function is as follows:

(1) $$Cross\;Entropy\;Loss=-\sum_{i}^{C}y_{i}\log\left(p_{i}\right)$$

where $y_{i}$ is the one-hot coded vector of true categories and C is the total number of categories, $y_{i}=1$ when the sample belongs to category i, otherwise $y_{i}=0.$ $p_{i}$ is the vector of probability distributions predicted by the model. Σ denotes summation over all categories.

#### 2.3.2. Weighting Design

In the standard cross-entropy loss function, all classes are given the same weight by the model. However, in the case of unbalanced category distribution, this treatment may lead to the dominant role of the majority class samples weakening the model’s ability to learn from the minority classes, which in turn masks the contribution of the minority classes in the loss function. To address this problem, this paper designs an MAAL method to balance the impact of the majority and minority class samples on model training by dynamically adjusting the error weights of different classes. Specifically, the method first calculates the error rate of each category to measure the prediction ability of the model on different categories. The expression for calculating the category error rate is as follows:

(2) $${class\;error\;rates}_{i}=\frac{{class\;error}_{i}}{\sum_{j=1}^{C}{class\;error}_{j}}$$

where ${class\;error\;rates}_{i}$ denotes the error rate for category i, C is the total number of categories, class error is the number of erroneous samples in each category, and Σ denotes the sum of misclassifications in all categories.

However, since the category error rate is usually distributed in the interval [0,1], its adjustment range is more limited, and it is difficult to fully alleviate the category imbalance problem. For this reason, this paper exponentially amplifies the category error rate to enhance its influence on a few category samples and make the weight adjustment more significant. The improved weight calculation formula is as follows:

(3) $$w_{i}=e^{\left({class\;error\;rates}_{i}\right)}$$

where $e^{x}$ is the exponential function and $w_{i}$ denotes the weight of the class i. This method can significantly increase the weight of the samples in the wrong class, thus reinforcing the model’s focus on the minority class.

#### 2.3.3. Weighted Cross Entropy Loss Function

**Figure 4:**
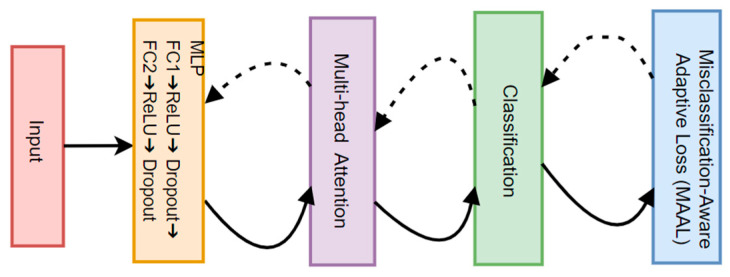
Flow of the MAAL algorithm running in the primary model.

After completing the adaptive adjustment of weights, this paper introduces the weighting factor based on the traditional cross-entropy loss function to form a weighted cross-entropy loss function, i.e., the MAAL method, so that the samples of different categories can adaptively adjust the contribution to the loss. Its specific formula is as follows:

(4) $$MAAL=-\sum_{i}^{C}y_{i}w_{i}log\left(p_{i}\right)$$

where $w_{i}$ denotes the weights calculated based on the error rate of each category. As shown in Figure 4, solid arrows indicate the forward propagation process. Dashed arrows indicate the backpropagation process. The MAAL algorithm obtains stronger gradient feedback by assigning higher weights to difficult-to-categorize samples in the backpropagation stage, thus directing the multi-attention mechanism to pay more attention to the key features of the misclassified samples, enhancing the aggregation within categories, and at the same time improving the MLP’s ability to fit complex category boundaries and reducing the dominant role of majority class samples on the loss function, thus improving the model’s learning ability for minority class samples.

### 2.4. Multi-Head Self-Attention Mechanism

In this paper, we introduce the multi-head self-attention mechanism (MHSA) in the model optimization process to enhance the feature extraction capability and the overall performance of the model. The multi-head self-attention mechanism is a widely used technique for deep learning, especially in natural language processing (NLP) and computer vision (CV) tasks. Its core idea is to capture multi-level long-range dependencies within the data by computing multiple self-attention modules in parallel to enhance the expressiveness and flexibility of the model.

In the multi-attention mechanism, the input vectors are mapped into multiple sets of query, key, and value matrices, and each set of matrices computes the attention weights separately to capture the feature relationships in different subspaces. The results of each set of attention are linearly transformed and then stitched together as the final output. The specific formula is as follows:

(5) $$MultiHead\left(Q,K,V\right)=Concat\left({head}_{1},{head}_{2},\ldots ,{head}_{h}\right)W^{O}$$

where each attention head is calculated as follows:

(6) $${head}_{i}=Attention\left({QW}_{i}^{Q},{KW}_{i}^{K},{VW}_{i}^{V}\right)$$

and each single-head attention is calculated as follows:

(7) $$Attention\left(Q,K,V\right)=Softmax\left(\frac{{QK}^{T}}{\sqrt{d_{K}}}\right)V$$

$W_{i}^{Q}\in R^{d_{model}\times d_{k}}$, $W_{i}^{K}\in R^{d_{model}\times d_{k}}$, and $W_{i}^{V}\in R^{d_{model}\times d_{v}}$ are head-specific projection matrices where the respective dimensions are set ($d_{model}=64$, h=4, $d_{k}=d_{v}=16$), $W^{O}$ is the output projection matrix, and $d_{K}$ is the dimension of the key, which is used to scale the dot product values to stabilize the gradient. Equation (5) defines the overall output form of the multi-head attention mechanism, which is obtained by splicing the computational results of multiple independent attention heads and performing a linear transformation; Equation (6) describes the specific computational process of each attention head, where the inputs are fed into the attention function after a linear projection; and Equation (7) shows that the weight computation based on the scaled dot product and weighted summation operation is the realization of the information extraction of each attention head the core step to realize the information extraction from each attention head.

The advantage of the multi-head self-attention mechanism is that it can capture the correlation between features from different perspectives to learn the complex patterns of high-dimensional data more comprehensively. In this paper, we combine the multi-head self-attention mechanism with the MLP model to enable it to compute multi-level correlations among features, focusing on local and global dependencies. In addition, the MAAL algorithm, by combining the multi-head self-attention mechanism, not only significantly improves the classification performance for a small number of classes but also enhances the robustness of the model to data distribution bias and noise interference, thus effectively mitigating the category imbalance problem.

### 2.5. Error Sample Management Mechanism

**Figure 5:**
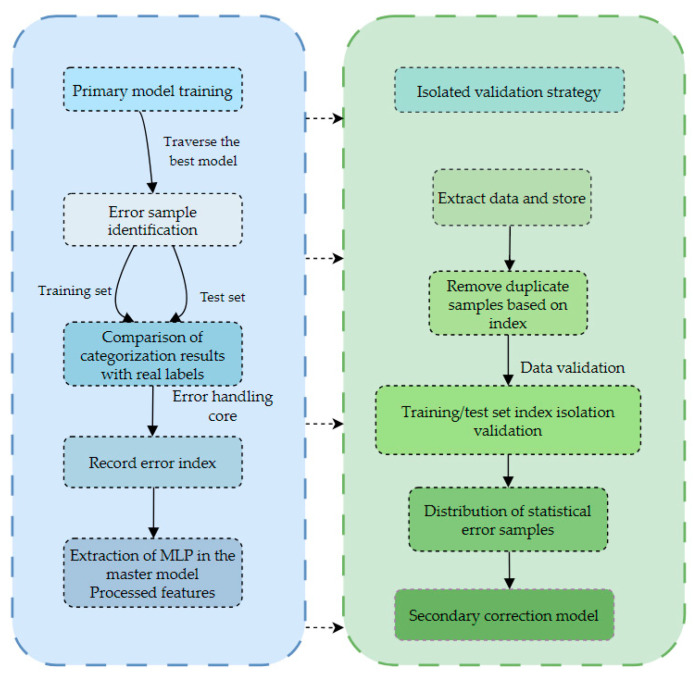
Error sample management mechanism flowchart.

As shown in Figure 5, the error sample management mechanism has the following two main aspects. To prevent interference with the overall model performance during the extraction of erroneous data, this paper designs a refined error sample extraction strategy. The original row number indexes of the data are saved during the process of data partitioning; during the iterative training process of the primary model, the best performing model is saved each time; finally, in the error sample extraction stage, all of the saved best models are traversed, the indexes of the error samples in the training set and the test set in each of the best models are recorded, and the samples that the MLP has processed are screened according to these indexes. With this strategy, it is ensured that the extracted error samples are all derived from the data misclassified by the primary model, which is used as input to the secondary correction model.

For the problem of data leakage protection, this paper introduces a data index isolation mechanism to fundamentally eliminate the risk of data leakage. This mechanism indexes and de-weights the extracted data to ensure that there is no overlap between the training set and the test set, thus avoiding the possibility of test data mixing in the training data. Through this mechanism, one can effectively avoid the phenomenon of false accuracy and model overfitting caused by data leakage. In addition, this mechanism is especially important in medical application scenarios.

Through the above strategies, this paper effectively avoids the possible misleading effects of data leakage while strengthening the model’s learning ability for difficult-to-classify samples, ensuring the safety and reliability of the model in medical applications.

## 3. Experiment

### 3.1. Important Parameters and Computational Efficiency

All model training and inference are undertaken in an environment equipped with an Intel i5-8300H, CPU (4 cores/8 threads at 2.30 GHz; Intel Corporation, Santa Clara, CA, USA). Model development and training were carried out using PyTorch version 1.8.0+cpu (PyTorch—Facebook AI Research, Menlo Park, CA, USA). During the training process, the model uses the AdamW optimizer, with a learning rate of 0.001, a batch size is set to 32, and a total of 600 epochs are trained for the primary model; the secondary correction model is trained for 1000 epochs. The entire training process takes 354.6 s in total, and the peak memory occupies about 172 MB. An early stopping mechanism is implemented during training, the patience value is set to 50, and L2 regularization (1 × 10^−5^) is used to prevent overfitting.

All hyperparameters are determined manually by multiple experiments to ensure that the model has better generalization capabilities. In the MAAL dynamic weighting strategy, we do not update the weights for the first 60 epochs, aiming at letting the model complete autonomous feature learning and achieve initial stabilization before intervening in the weighting to obtain the best results. To this end, we conducted comparative experiments at 30, 60, and 90 epochs, and the results show that 60 epochs have the best performance, so we adopt them. At the same time, to prevent the imbalance of the model due to the high error rate of certain categories, we limit the dynamic weights to the range of [1,5]. In the parameter tuning of the self-attention mechanism, after testing the input dimensions in 32, 64, and 128 settings, 64 dimensions performs the best; the number of attention heads is compared in 2, 4, and 8 heads, and 4 heads is the most effective; the dropout ratio is 0.5 after three attempts of 0.3, 0.5, and 0.7, and 0.5 is the most robust.

### 3.2. Dataset

The dataset chosen for this study is the prenatal fetal contraction monitoring (CTG) dataset from the University of California, Irvine (UCI) Machine Learning Knowledgebase. This is one of the most popular databases used by researchers of intelligent classification of fetal heart rate monitoring and contains 2126 feature records extracted from ECGs, with each record covering 21 CTG features. These records were subsequently classified and labeled by three obstetricians into three categories: normal: 0, suspicious: 1, and abnormal: 2.

### 3.3. Feature Preprocessing

#### 3.3.1. Feature Selection

To enhance the classification performance of the model, this paper adopts the minimum redundancy maximum relevance (mRMR) [38] feature selection method in the feature preprocessing stage. The mRMR method maximizes the retention of the information that has the most classification ability for the classification task by filtering the features that have the maximum relevance to the target variables with the minimum redundancy, while effectively reducing the data dimensionality. Ultimately, eight key features are selected in this paper to prepare for subsequent modeling and model optimization.

#### 3.3.2. Data Division

In this paper, we utilize scikit-learn’s train_test_split function, setting stratify = y (i.e., employing stratified sampling based on the proportion of true labels) and a fixed random seed (random_state = 42) to partition all samples into a training set and a test set in a 77%/23% ratio. This ensures that the ratio of each category in the training set to the test set remains consistent, thereby enhancing the reproducibility of the experiments. This division is applied during the primary model stage. In the secondary correction model, we directly use the erroneous samples from the training process of the primary model as the training data for the secondary correction model, while the erroneous samples identified during the test phase of the primary model serve as the test data of the secondary correction model, with the specific distribution shown in Table 2. To avoid the test set error samples in the primary model being divided into the training set error samples, this paper implements an error sample management mechanism.

#### 3.3.3. Normalization Methods

The model uses the Stander scaler method to normalize the data, specifically by varying each eigenvalue x with the following formula:

(8) $$x_{new}=\frac{x-\mu }{\sigma }$$

where μ is the mean of the feature and σ is the standard deviation of the feature. The purpose is to eliminate the influence of different feature magnitudes on the model, so that all features are in the same magnitude, so that, in turn, the attention weight calculation is fairer and does not produce bias.

### 3.4. Evaluation Criteria:

To evaluate the model performance, the most common evaluation criteria are used in this paper to measure the accuracy, precision, recall, F1-score, and AUC of the adjusted misclassification model. The following are the specific definitions and formulas of these evaluation criteria.

#### 3.4.1. Accuracy

Accuracy is the most basic performance evaluation criterion and is used to measure the proportion of samples correctly predicted by the model as a percentage of the total samples. The formula is as follows:

(9) $$Accuracy=\frac{TP+TN}{TP+TN+FP+FN}$$

#### 3.4.2. Precision

Precision measures the proportion of samples predicted by the model to be in the positive category that are actually in the positive category, i.e., the accuracy of the prediction results, and a model with a high degree of precision can help doctors more accurately identify patients with the disease, and thus designate a more effective treatment plan. The formula is as follows:

(10) $$Precision=\frac{TP}{TP+FP}$$

#### 3.4.3. Recall

Recall measures the proportion of all samples that are actually in the positive category that are correctly predicted by the model to be in the positive category. In medical diagnosis, the consequences of a missed diagnosis are more serious than a misdiagnosis, because a missed diagnosis may lead to a worsening of the condition or missing the best time to treat it. The leakage rate of the model can be known by observing the recall value. The formula is as follows:

(11) $$Recall=\frac{TP}{TP+FN}$$

#### 3.4.4. F1-Score

F1-score is able to combine precision and recall, aiming to balance the relationship between the two for a more comprehensive evaluation of model performance. In the case of unbalanced data, the precision rate may mislead the evaluation performance of the model, while the F1-score is able to better respond to the performance of the model on a small number of classes. Its formula is as follows:

(12) $$F1\text{-Score}=2*\frac{Precision*Recall}{Precision+Recall}$$

#### 3.4.5. Area Under the ROC Curve (AUC)

AUC measures the ability of the model to correctly rank randomly selected pairs of positive and negative samples, i.e., the predicted probability of a positive sample is higher than the probability of a negative sample. AUC is the area under the ROC curve, and the ROC curve expresses the relationship between the true rate of true cases (TPR) and the false positive rate of false positive cases (FPR). The closer the value of AUC is to 1, the higher the diagnostic accuracy of the model. The formula is as follows:

(13) $$TPR\left(True\;Positive\;Rate\right):TPR=\frac{TP}{TP+FN}$$

(14) $$FPR\left(False\;Positive\;Rate\right):FPR=\frac{FP}{FP+TN}$$

where:

TP (true positive): the number of positive classes predicted by the model and actually positive.
TN (true negative): the number of negative classes predicted by the model that are actually negative.
FP (false positive): the number of cases where the model predicts a positive class but the actual class is negative.
FN (false negative): the number of cases where the model predicts a negative class but it is actually a positive class.

### 3.5. 6 Bootstrap 95% Confidence Intervals

Bootstrap 95% confidence intervals are calculated by resampling the test set multiple times with putbacks, calculating the metrics of interest on each bootstrap sample (1000 times), and then taking the 2nd and 97.5th percentiles of these estimates as upper and lower bounds for the interval. This non-parametric approach requires no distributional assumptions, is suitable for complex indicators, and produces an interval whose width reflects the stability of the indicator—the narrower the interval, the more reliable the performance estimate.

### 3.6. Ablation Experiments

**Figure 6:**
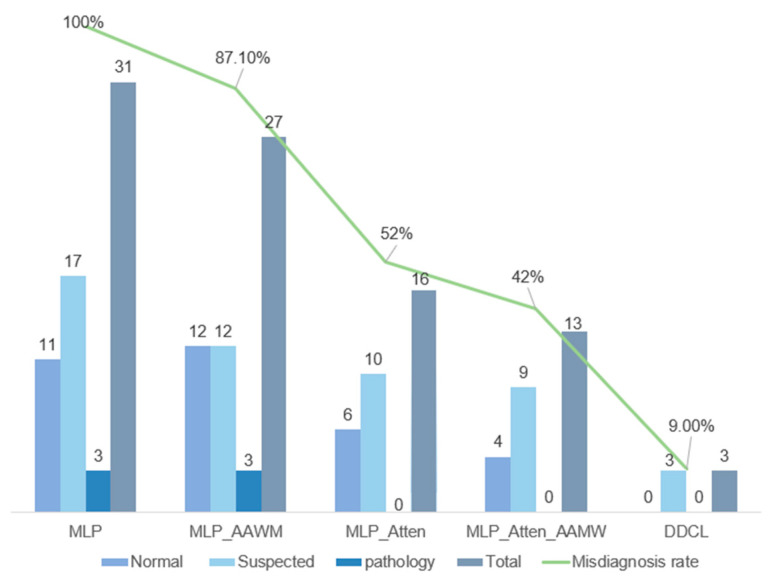
Comparison of error data and misdiagnosis rate for different categories in each model is shown.

This ablation experiment system disentangles the contribution of each component to the model performance (see Table 3). The baseline MLP model achieved 93.66% accuracy with an AUC value of 0.9766 without introducing any optimization strategy, validating the effectiveness of the deep learning network in capturing the nonlinear relationship of fetal health features. Although it reaches a weighted F1-score of 0.9358, this result is largely driven by the overrepresented “Normal” class, masking poor performance on minority classes. As shown in Figure 6, the misclassification rate reaches 23% for the “Suspect” class and 10% for the “Pathological” class, while the normal class has 6–9 times more samples—indicating that class imbalance remains significant. Introducing adaptive MAAL improves overall performance without data resampling: precision and recall both increase by 0.93%, showing that MAAL effectively addresses imbalance by learning from sample errors. With the addition of the multi-head self-attention mechanism (MLP-Attn), accuracy rises by 3.07 percentage points to 96.73%, and AUC increases to 0.9854, confirming that attention enhances the model’s focus on key features and improves its discriminative power.

When MAAL synergizes with the attention mechanism (MLP-Attn-MAAL), the model exhibits a positive coupling effect between components: the F1-score improves by 3.73 percentage points from the baseline and the standard deviation of the AUC fluctuation decreases from 0.0027 to 0.0015, which further verifies the optimization of the complementarity between the dynamic weights and the attention mechanism, and also indicates that the model is stable and reliable. Finally, MAC-NET learns the error samples of the primary model through the secondary correction model, driving the model to break through the performance bottleneck and achieve 99.39% accuracy with a high AUC value of 0.9983. This result fully proves that the model has excellent performance in solving the category imbalance problem and correcting the error samples.

In this experiment, 1000 bootstrap resamplings based on the F1-score are conducted to assess the significance of performance differences between MAC-NET and each ablation model. All 95% confidence interval (CI) lower bounds of ΔF1 are significantly above zero, confirming that MAC-NET consistently and significantly outperforms the baselines across different data splits. Moreover, the progressive narrowing of ΔF1 CIs—from the base model (MLP) to the more advanced variants (e.g., MLP-Attn-MAAL)—highlights both the increasing performance gains and the improved stability introduced by the proposed components, further validating their effectiveness and robustness.

### 3.7. Comparison Experiments:

#### 3.7.1. Comparison of Methods for Handling Unbalanced Data

To verify the effectiveness of the MAAL algorithm proposed in this paper in dealing with the category imbalance problem, this paper introduces the focal loss and SMOTE methods; carries out the comparison experiments for the two baseline models of MLP and XGBoost, respectively, in the following; and evaluates their classification performances on different categories, with the results of the experiments are shown in Table 4. Focal loss is a loss function designed to solve the problem of category imbalance. Its core introduces a moderating factor on top of the standard cross-entropy loss. When the model has a high prediction probability for a category (i.e., easily categorized samples), the adjustment factor reduces its weight, thus reducing the contribution of these samples to the total loss; on the contrary, for difficult samples or misclassified samples, the loss weight will increase. Therefore, focal loss can effectively focus on difficult samples and improve the model’s ability to learn a few categories. The formula for focal loss is as follows:

(15) $$FL\left(p_{t}\right)=-\alpha {\left(1-p_{t}\right)}^{\gamma }\log\left(p_{t}\right)$$

where α is a balancing factor, used to adjust the category weights in this paper, set to 0.25; γ is an adjustment parameter, which controls the degree of focusing of the difficulty samples, this study uses γ = 2; ${\left(1-p_{t}\right)}^{\gamma }$ is a difficulty weighting factor; and $p_{t}$ denotes the predicted probability of the correct category.

The synthetic minority oversampling technique (SMOTE) is a classical category imbalance processing method, whose core idea is to artificially synthesize minority class samples through feature space interpolation. The specific implementation process is as follows:

Neighborhood sampling: for each minority class sample $x_{i}$, calculate its k-nearest neighbors (k = 5) in the feature space.

Linear interpolation: randomly select the neighborhood sample $x_{i}$ to generate a new sample, as follows:

(16) $$x_{new}=x_{i}+\lambda \cdot \left(x_{j}-x_{i}\right)$$

where λ ∈ [0,1] is the random interpolation coefficient and $x_{j}$ is a randomly selected neighbor from the k nearest neighbors of $x_{i}$.

Sample expansion: repeat the above process until the sample size of each category is equalized.

Experiments show that the MLP family of methods significantly outperforms the XGBoost family of models in medical data imbalance processing. Among these, the XGBoost model performs weakly in the suspected class, achieving a recall of only 0.6912. Even when combined with focal loss for improvement, the recall of the suspected class is only slightly improved, whereas MLP-Attn-MAAL significantly improves the recall of the suspected class to 0.88 by the combination of MAAL with a multi-head self-attention mechanism, which is much more efficient and effective than that of the focal loss’s limitation of relying on static weight adjustment. MAAL optimizes the weight allocation by dynamically sensing the change of sample error rate, thus adjusting the model learning direction more precisely. In the pathology class task, the AUC of MLP-FL is 0.9914, while MLP- Attn-MAAL improves to 0.9995. Although XGb-SMOTE achieves an AUC of 0.9921 in the pathology class, its recall of 0.9605 for the normal class is still significantly lower than that of the MAC-NET recall value for the normal and suspected classes.

MAC-NET achieves 100% accurate recognition of both normal and pathological cases in the test set through a secondary correction model. The first-stage primary model (MLP-Attn-MAAL) uses the attention mechanism to enhance the features of a few categories, such as the pathological category, so that the recall value of the pathological category reaches 1, i.e., zero missed diagnosis is achieved in the test set; the second-stage secondary correction model performs targeted corrections for the erroneous samples of the primary model. The MAC-NET achieves an AUC of 1 for all the categories through the synergistic optimization of the two-stage MAAL and the recall values of both the normal and suspected categories are greatly improved. While some categories reached 100% accuracy in the test set, caution is warranted regarding the potential risk of overfitting. Notably, the 95% confidence intervals in Table 5 indicate that the lower bounds are very close to MAC-NET’s point estimates, and the overall intervals are considerably narrower, suggesting that the improvement in model performance is statistically robust and not a result of overfitting.

#### 3.7.2. Comparison of the Number of Error Samples

As shown in Figure 6, the bars represent the number of misclassified samples in each category. The baseline MLP model incurred three errors in the pathology category, whereas MAC-NET eliminated all pathology errors—effectively reducing those three cases to zero. Although the MLP-Atten-MAAL primary model also achieved zero pathology errors, it still accumulated thirteen errors overall (four in the normal category and nine in the suspected category), demonstrating that a single-stage classifier struggles to balance performance across multiple classes.

By contrast, MAC-NET’s two-stage correction framework inherits the primary model’s flawless pathology predictions and then further refines the classification of the normal and suspected classes. Specifically, it cuts the suspected-class errors from nine down to three and brings the normal-class misclassifications to zero, yielding a substantial reduction in overall misdiagnoses.

To quantify this improvement, we treat the MLP model’s misdiagnosis count as the 100% reference point. Introducing MAAL alone or the attention mechanism alone reduces the misdiagnosis rate to 87.1% and 52%, respectively, relative to MLP. When both techniques are combined in the primary model, the rate falls further to 42%. Finally, MAC-NET drives the misdiagnosis rate down to just 9% of the baseline (i.e., a 91% reduction compared with MLP), clearly highlighting its corrective power.

These results demonstrate that MAC-NET not only mitigates the risk of local overfitting in multi-category classification tasks but also markedly boosts accuracy on boundary-ambiguous samples. This two-stage approach therefore offers greater reliability for clinical diagnosis, effectively overcoming the limitations inherent in traditional single-stage models when dealing with complex medical data.

#### 3.7.3. Experiments on the Generalization Ability of the MAAL Algorithm

To comprehensively assess the generalizability of the model, two publicly available multi-category unbalanced datasets, which are available from the KEEL repository, are selected for comparison in the experimental study. To ensure the reliability of the experimental assessment, the datasets are preprocessed in this paper by removing the categories with fewer than 10 total samples. The reason for this is that the experiment is divided using a 77% training set and a 23% test set. If the number of samples in a category is less than 10, the test set can only contain a maximum of 2 test samples of that category, which may lead to unstable evaluation results, thus affecting the comparison of model performance. The following is a brief description of the dataset used in this paper.

The balance dataset simulates a physical balance problem, containing 625 samples, each representing a combination of weight and distance between the left and right sides of the balance, to predict the state of the balance (left-tilted, right-tilted, or balanced). It contains four continuous-type features: left-weight (left side weight), left-distance (left side distance), right-weight (right side weight), and right-distance (right side distance). The data are labeled with three categories: left tilt (L), right tilt (R), and balanced (B), with a category distribution of 288:49:288. Due to the severe imbalance of the data, this dataset is often used to study multicategorical imbalance problems and is ideal for evaluating the performance of classifiers on imbalanced datasets.

The page block dataset comes from the analysis of document page blocks; the goal is to categorize the content blocks in the page into different types such as body, title, picture, etc. The data contain 5473 samples and 10 continuous-type features. The features mainly describe the geometric and positional characteristics of the page blocks, such as width, height, area, etc. The data are divided into five categories: 1 (body text), 2 (title), 3 (picture), 4 (horizontal line), and 5 (digitized object), of which the body text category accounts for about 89%, the remaining categories have a smaller amount of data, and there is a significant imbalance. Due to the removal of the category numbers that are less than 10, the data are left with only three categories: body text, title, and digitized object. The distribution of the categories is 492:33:12. This dataset is widely used in the study of unbalanced categorization tasks.

To verify the applicability of the MAAL algorithm and MAC-NET proposed in this paper in unbalanced data scenarios. In this paper, three datasets with significant category imbalance characteristics, balance, page block, and fetal health, are selected and compared with various canonical machine learning algorithms (e.g., decision trees, support vector machines, logistic regression, random forests, etc.) as well as multilayer perceptron machines (MLPs), and the experimental results are shown in Table 5.

All three datasets suffer from extreme category imbalance: the balance dataset has only 7.84% of right-skewed categories, the page block dataset has 0.22% of digitized object categories, and the fetal health dataset has only 8% of pathology categories. In this extremely unbalanced scenario, the MLP-Attn-MAAL model proposed in this paper still maintains high accuracy with 97.92%, 97.58%, and 97.34% in the above three datasets, respectively. Compared with the traditional machine learning decision tree model (DT), the accuracy of MLP-Attn-MAAL in the balance dataset is improved by 31.26 percentage points, which verifies the effectiveness of the adaptive weighting mechanism in synergistically optimizing the dominant and tail classes.

On this basis, MAC-NET further breaks through the performance bottleneck and improves the model’s adaptability through the secondary correction model. The accuracy in the three unbalanced datasets reaches 99.39%, 99.31%, and 99.19%, respectively, all of which are close to perfect classification. Crucially, 95% bootstrap confidence intervals (1000 resamples) for MAC-NET all exceed 0.965 and are notably narrower than those of other models, underscoring both the statistical significance and consistency of its gains. This demonstrates that MAC-NET not only breaks through the performance ceiling of single-stage models but also delivers highly reliable classification by hierarchically correcting errors without prior class information—making it a standout solution for complex, imbalanced classification tasks.

### 3.8. Model Visualization

#### 3.8.1. Display of Weight Changes

**Figure 7:**
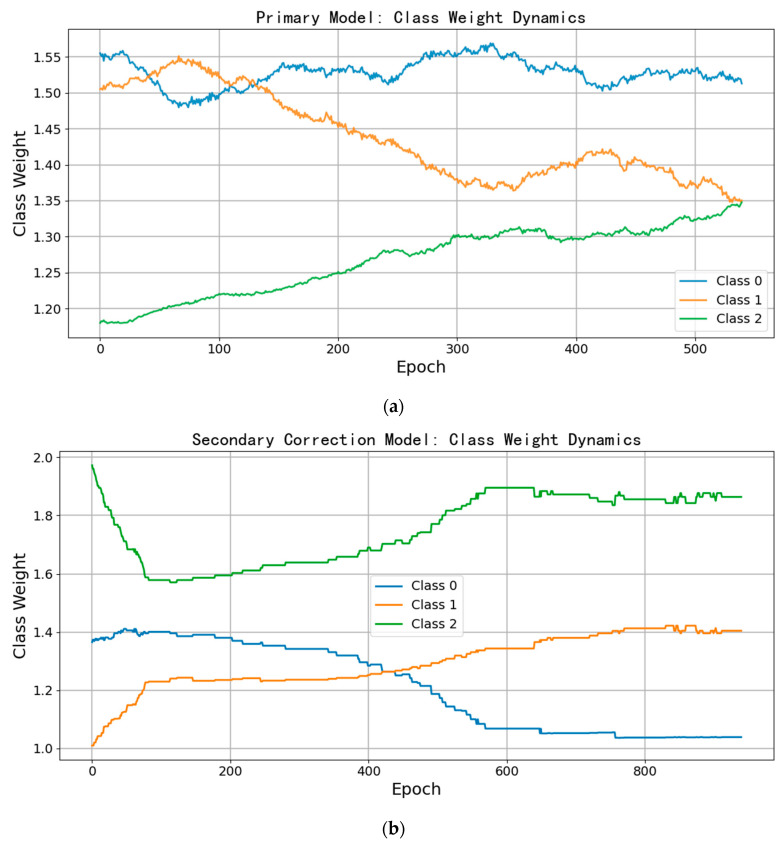
MAAL weight change curve. (**a**) The primary model dynamic weight change curve. (**b**) The secondary correction model weight change curve.

The primary and secondary correction models in MAC-NET delay adaptive weight updates for the first 60 iterations to allow autonomous feature learning; accordingly, Figure 7a omits those rounds. During primary training, MAAL’s dynamic weighted loss—driven by category error rates—first compensates for the hardest class (pathology, category 2) by boosting its weight, which then gradually stabilizes as the model learns its discriminative features. Meanwhile, the suspicious class (category 1) exhibits large early fluctuations—reflecting its ambiguous features—before its weight settles downward as accuracy improves; the normal class (category 0) maintains a relatively high but gradually decreasing weight, indicating balanced learning across all three categories. These trends confirm MAAL’s effectiveness at dynamically adjusting to class-specific learning difficulty and stabilizing the model under severe imbalance.

In the secondary correction stage (Figure 7b), the extracted error set still presents some imbalance (Table 2), so MAAL is reapplied. With a smaller, more balanced dataset, weight adjustments converge more rapidly: the pathology weight initially drops sharply—drawing focus due to its high error rate—then rebounds as features are learned; the suspicious class, now the majority, sees its weight rise early before stabilizing; the normal class weight briefly increases and then declines to a steady level.

Overall, the secondary model’s weight curves are much smoother than those of the primary model because it only corrects misclassified samples—simplifying sample competition and reducing fluctuation. Figure 7a showcases MAAL’s dynamic weight adjustments—not model convergence—and these fluctuations are expected as it fine-tunes weights across a large, imbalanced training set for optimal accuracy. In contrast, Figure 7b appears smoother because the secondary model operates on a smaller, more balanced dataset, finding effective weights more rapidly. This reflects the dataset scale, not a flaw in the primary model.

#### 3.8.2. t-SNE Diagram Presentation

**Figure 8:**
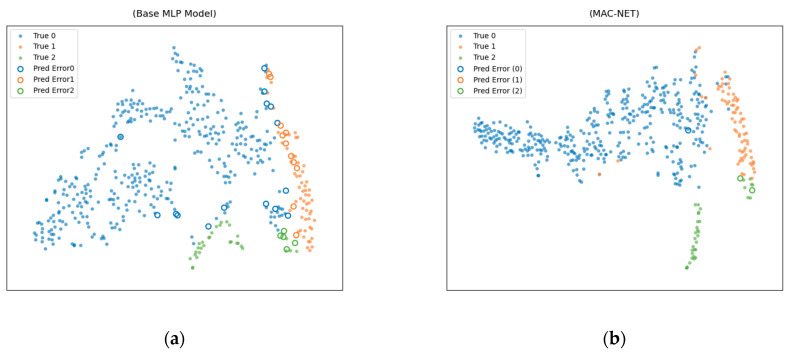
t-SNE (perplexity = 30, iterations = 1000) visualization. (**a**) Baseline model of the MLP model. (**b**) t-SNE plot of the MAC-NET model.

In this study, t-SNE is used for visualization and analysis to demonstrate the optimization effect of the MAC-NET model in feature space. Solid circles indicate the samples that are correctly classified by the model, and hollow circles of different colors indicate that the model misclassifies the samples into the category corresponding to that color. Figure 8a shows that the normal class samples (blue) of the baseline MLP model show a diffuse distribution, which partially intrudes into the region of the suspected class (orange) and the pathological class (green), and the pathological class samples are loosely distributed in the lower-right quadrant and have a significant overlapping portion with the suspected class (orange-green mixing zone), resulting in blurred decision boundaries.

In Figure 8b, the MAC-NET framework clusters the feature space through the dynamic weighted fusion attention mechanism, with the normal class (blue) forming a high-density core cluster whose distribution shrinks by about 40%, the pathology class (green) aggregating into a compact single-peak structure in the lower right, and the suspected class samples (orange) shifting to the upper-right quadrant of the feature space to form an independent aggregation area. Compared with the baseline model, the area of the orange-green overlap region of the MAC-NET architecture is significantly reduced, and the boundaries between the three categories of sample clusters are clearer, indicating that the model has been significantly improved in category differentiation ability.

To quantify these results, as shown in Table 6,we calculated the silhouette coefficient (the closer to 1 the better) and the Davis–Boldin index (the closer to 0 the better). The silhouette coefficient of 0.2008 for MAC-NET is 50.6% higher than that of the MLP at 0.1333, and the Davis–Boldin index at 0.8983 is 16.5% lower than that of the MLP at 1.0758. These data confirm that the feature space learned by MAC-NET has tighter intra-class cohesion and stronger inter-class separation, which reduces overlap and makes the decision boundary clearer, which is consistent with the effect of t-SNE visualization.

### 3.9. Comparative Analysis with Other Existing Studies

As shown in Table 7, the performance and methods of the MAC-NET framework proposed in this paper are analyzed in comparison with other models. Compared with other models, the advantage of MAC-NET is that it pays full attention to the problem of category imbalance without going through the method of increasing samples, such as sampling. It has a high accuracy rate of 99.39% without introducing additional noise.

## 4. Discussion

In this study, to address the problems of classification bias, low recognition rate of a few classes, and high misdiagnosis rate caused by the imbalance of CTG data categories in the auxiliary diagnosis of fetal health, we propose a main-auxiliary correction network (MAC-NET) based on the method of misclassification-aware adaptive loss (MAAL) and the secondary correction model. The experimental results show that the method has significant advantages in alleviating data imbalance, improving the recognition of pathological and suspected classes, and reducing the risk of misdiagnosis and omission.

As shown in Table 7, compared with static balancing strategies such as oversampling [24], SMOTE [17], and focal loss [29], MAC-NET can make fine corrections for boundary fuzzy samples through MAAL dynamic weighting with multiple self-attention, combined with a secondary correction mechanism. Compared with other machine learning models [2], Light GBM [40] and other models in the model accuracy improvement are more obvious. The model also outperforms other methods on multiple unbalanced datasets. A total of 1000 bootstrap confidence interval analyses also verify the stability of the performance improvement. The two-stage architecture effectively breaks the convergence bottleneck of the single-stage model, significantly suppresses the fluctuation of most class weights, and enhances the generalization potential.

However, this study is still limited by the size and heterogeneity of the public dataset and needs to be validated with large samples from multiple centers, the model complexity and computational overhead also need to be evaluated in real environments, and the interpretability of the deep features, although highly separable, needs to be enhanced by SHAP, LIME, and other methods. The method in this paper achieved excellent performance in the experiment, but there are still some limitations. The weight change of the dynamic weighted loss function fluctuates greatly, and a more robust adaptive mechanism can be further explored in future work to make it smoother.

To realize the application of “human-machine cooperation,” MAC-NET can be embedded into a CTG monitoring system to receive data from the fetal heart monitor in real time and output the risk level and confidence interval. When the model detects a high-risk sample (e.g., pathology), it will immediately trigger an alert and highlight the visual decision boundary on the interface, helping doctors to quickly locate abnormal waveforms and then make decisions.

## Figures and Tables

**Table 1:** Model parameter configuration.

Model	Parameter	Value
Primary model	Fc1 layer	(8,64)
	Dropout1/2	0.3
	Fc2 layer	(64,64)
	Attention layer	4 head
	Classification layer	(64,3)
Secondary correction model	Fc1 layer	(64,32)
	Dropout	0.5
	Attention layer	4 head
	Attention-dropout	0.5
	Classification layer	(32,3)

**Table 2:** Distribution of categories at different stages.

Category Distribution	Normal	Suspect	Pathology
Primary model training set	1275	220	142
Primary model test set	380	75	34
Training set error samples	122	220	142
Test set error samples	37	75	34

**Table 3:** Results of ablation experiments by model.

Model	Accuracy	Precision	Recall	F1-score	AUC	95% CI
MLP	0.9366	0.9355	0.9366	0.9358	0.9766	[0.0379, 0.0802]
MLP-MAAL	0.9448	0.9450	0.9448	0.9448	0.9774	[0.0306, 0.0690]
MLP-Attn	0.9673	0.9674	0.9673	0.9671	0.9854	[0.0270, 0.0640]
MLP-Attn-MAAL	0.9734	0.9732	0.9734	0.9731	0.9858	[0.0227, 0.0575]
**MAC-NET**	**0.9939**	**0.9941**	**0.9939**	**0.9937**	**0.9983**	**-**

**Table 4:** Comparison of different algorithms for handling balanced data with XGboost.

Model	Recall			AUC		
	Normal	Suspect	Pathology	Normal	Suspect	Pathology
XGb	0.9711	0.6912	0.9250	0.9704	0.9509	0.9975
MLP	0.9711	0.7733	0.9118	0.9738	0.9629	0.9930
XGb-FL	0.9738	0.7059	0.9250	0.9623	0.9340	0.9965
MLP-FL	0.9711	0.8000	0.9412	0.9766	0.9679	0.9914
MLP-smote	0.9457	0.9391	0.9920	0.9904	0.9905	0.9980
XGb-smote	0.9605	0.9685	0.9921	0.9958	0.9969	1.0000
MLP-Attn-MAAL	0.9895	0.8800	1.0000	0.9825	0.9755	0.9995
MAC-NET	1.0000	0.9600	1.0000	1.0000	1.0000	1.0000

**Table 5:** Comparison of the accuracy of model generalization capabilities under different datasets.

Model	Fetal Health		Balance		Page Block	
	Acc	95% CI	Acc	95% CI	Acc	95% CI
DT	90.79%	[0.8834, 0.9325]	75.00%	[0.6804, 0.8194]	95.16%	[0.9113, 0.9839]
SVM	80.77%	[0.8057, 0.8712]	85.41%	[0.8542, 0.9514]	94.35%	[0.9194, 0.9919]
LG	80.98%	[0.8139, 0.8753]	85.42%	[0.8542, 0.9514]	94.35%	[0.9194, 0.9919]
RF	92.02%	[0.8998, 0.9448]	86.81%	[0.8125, 0.9168]	95.16%	[0.9113, 0.9839]
KNN	89.57%	[0.8793, 0.9305]	81.94%	[0.8194, 0.9306]	92.74%	[0.8790, 0.9677]
MLP	93.66%	[0.9141, 0.9591]	93.60%	[0.8958, 0.9722]	96.77%	[0.9355, 0.9919]
MLP-Atten-MAAL	97.34%	[0.9571, 0.9857]	97.92%	[0.9444, 0.9931]	97.58%	[0.9435, 1.0000]
**MAC-NET**	**99.39%**	**[0.9857, 1.0000]**	**99.31%**	**[0.9653, 1.0000]**	**99.19%**	**[0.9758, 1.0000]**

**Table 6:** t-SNE quantitative indicator.

Model	Silhouette Coefficient	Davies–Bouldin Index
MLP	0.1333	1.0758
MAC-NET	0.2008	0.8983

**Table 7:** Performance comparison with other existing models.

Author	Methodology	Accuracy
Chen Y et al. [16]	Deep forest(DF)	92.64%
Salini Y et al. [2]	ML models	93%
Cömert Z et al. [22]	Extreme learning machine (ELM)	93.42%
Abiyev R et al. [39]	Type-2 fuzzy neural networks	96.6%
Mandala S K et al. [40]	Light GBM	98.31%
Mushtaq G et al. [18]	Deep neural network (DNN)	99%
Al Duhayyim M et al. [24]	Oversampling and XGBoost, Light GBM, Cat Boost	99%
Bharadwaj K [17]	XGBoost and SMOTE	99%
Methodology of this paper	MAC-NET	99.39%

## Data Availability

This paper uses publicly available datasets. One of the fetal health datasets can be found here https://archive.ics.uci.edu/ml/datasets/cardiotocography (accessed on 21 March 2025). The imbalance comparison dataset is taken from KEEL: A software tool to assess evolutionary algorithms for data mining problems (regression, classification, clustering, pattern mining and so on).
